# Comparison of clinical outcomes between culture-negative and positive peritonitis in patients undergoing maintenance peritoneal dialysis: a prospective cohort study

**DOI:** 10.1186/s12882-023-03389-7

**Published:** 2023-11-17

**Authors:** Kittiphan Chienwichai, Sorawat Sangaew, Laksamee Chuachanpipat, Arunchai Chang

**Affiliations:** 1 Division of Nephrology, Department of Internal Medicine, Hatyai Hospital, Songkhla, Thailand; 2 Department of Social Medicine, Hatyai Hospital, Songkhla, Thailand; 3 Department of Internal Medicine, Hatyai Hospital, Songkhla, Thailand

**Keywords:** Culture-negative, Peritoneal dialysis, Peritonitis, Treatment outcome

## Abstract

**Background:**

Culture-negative peritonitis is a serious complication in patients undergoing maintenance peritoneal dialysis (PD) and occurs in up to 40% of all peritonitis episodes. Despite its high incidence, data regarding treatment response and prognosis remain poorly defined. This study compared the clinical outcomes of patients with culture-negative and positive peritonitis.

**Method:**

This prospective cohort study was conducted between 2021 and 2022. Patients treated with maintenance PD who developed PD-associated peritonitis were included and received standard treatment. The primary endpoint was the primary response (resolution of peritonitis 10 days after the initiation of treatment).

**Results:**

Of the 81 patients who developed PD-associated peritonitis during the study, 35 and 46 had culture-negative and culture-positive peritonitis, respectively. Overall, 52 (64.2%) patients achieved the primary response: 24 (68.6%) in the culture-negative group and 28 (60.9%) in the culture-positive group (p = 0.630). There were no significant differences between the two groups in the incidence of complete cure (complete resolution of peritonitis without the need for Tenckhoff catheter removal or salvage antibiotic therapy or peritonitis within 120 days after treatment) (culture-negative vs. culture-positive group, 57.1% vs. 45.7%), refractory peritonitis (28.6% vs. 41.3%), relapse peritonitis (8.6% vs. 2.2%), repeat peritonitis (11.4% vs. 10.9%), salvage antibiotics (40.0% vs. 50.0%), permanent hemodialysis transfer (11.4% vs. 10.9%), Tenckhoff catheter removal (25.7% vs. 41.3%), or mortality (2.9% vs. 2.2%) (all p > 0.05).

**Conclusion:**

This study offers valuable insights into the clinical outcomes of culture-negative peritonitis versus culture-positive peritonitis. However, caution must be exercised in interpreting these findings due to the limitations of the small sample size.

**Clinical trial registration:**

The study was retrospectively registered in the Thai Clinical Trials Registry (TCTR20221130006).

**Supplementary Information:**

The online version contains supplementary material available at 10.1186/s12882-023-03389-7.

## Background

Peritoneal dialysis (PD)-associated peritonitis is a common and serious complication of PD [1], which accounts for 2–15% of mortality risk [2–5], 16–20% of cases of catheter removal [6, 7], and 16–18% of cases of transfer to hemodialysis [6, 8]. PD culture results influence the choice of antibiotics, treatment duration, and prognosis. The International Society for Peritoneal Dialysis (ISPD) guidelines recommend that the proportion of culture-negative peritonitis to be < 15% of all peritonitis episodes [9]. However, culture-negative peritonitis has been reported in up to 40% of all peritonitis episodes and remains a significant problem [9].

The clinical outcomes of culture-negative peritonitis are controversial, with conflicting data reported in the literature. However, many patients with this condition generally exhibit more favorable clinical outcomes than those with culture-positive peritonitis [10–12]. The cause of this more benign course is not yet fully understood. While empirical treatment for culture-negative peritonitis can pose challenges for clinicians, cause-specific treatment may have advantages such as facilitating the identification of potential sources of infection, which can lead to more targeted therapy [9, 13].

Our hypothesis was that the clinical outcome of culture-negative peritonitis is not different from, or may even be worse than, that of culture-positive peritonitis. Previous data on culture-negative peritonitis are mostly derived from retrospective cohort and registry studies. Therefore, we conducted a prospective cohort study at our center to compare the clinical outcomes of culture-negative and culture-positive peritonitis using more detailed data. In addition, we aimed to identify the culture practices associated with culture-negative peritonitis.

## Methods

All adult patients with PD who developed PD-associated peritonitis at Hatyai Hospital (a regional tertiary center in southern Thailand) between March 2021 and October 2022 were included in this study. The protocol was approved by the Institutional Review Board of Hatyai Hospital (HYH EC 42/2564), and the study was conducted according to the Declaration of Helsinki. Patients were required to be on PD for at least 3 months. The exclusion criteria for the study included individuals who had developed PD-associated peritonitis in the previous 120 days, pregnant individuals, those with secondary peritonitis (peritonitis from gastrointestinal source such as bowel perforation, abscess), and those who were already being treated with icodextrin. The study was retrospectively registered in the Thai Clinical Trials Registry (TCTR20221130006) on 30/11/2022. Written informed consent was obtained from all subjects before the commencement of the study.

Data were collected prospectively and included the following: baseline demographics, cause of the primary kidney disease, results of the laboratory tests taken on the day the patient developed PD-associated peritonitis, details of the person who performed PD, modalities of PD, peritoneal fluid white blood cell count, and the culture technique and procedure. For patients experiencing multiple episodes of peritonitis, only the initial episode was considered when comparing culture techniques and procedures between patients with culture-negative and culture-positive peritonitis, as well as for assessing primary and secondary culture-related outcomes.

Culture-positive peritonitis was diagnosed when at least two of the following were present: (1) clinical features of peritonitis (abdominal pain and/or cloudy dialysis effluent), (2) dialysis effluent white blood cell count > 100 µ/L with > 50% polymorphonuclear leukocytes, and (3) a positive dialysis effluent culture. Culture-negative peritonitis was diagnosed using criteria (1) and (2) above combined with a negative culture for any organism (including fungi and mycobacterium). We repeated peritoneal fluid cultures on day 3 when the culture remained negative with aerobic, mycobacterial, and fungal cultures. Our center uses the Bact/Alert automated blood culture system for every blood culture bottle processed. The agar plates used for PD fluid (PDF) culture included blood agar, MacConkey agar, and chocolate agar. For fungal culture, PDF was streaked onto Sabouraud dextrose agar and, if necessary, onto specific agar plates. Subsequently, it was incubated at both 25 and 37 °C for a duration of 14 days. For mycobacterial culture, PDF was inoculated into Lowenstein-Jensen medium slants and BACTEC MGIT 960 media, with incubation periods of 2 months and 42 days, respectively.

Due to the absence of established protocols for the PDF culture technique at our center so the selection of the PD collection and culture technique was at the discretion of the attending physicians and nurses. It is important to note that PD specimen collection for culture was the responsibility of the nurse working at the specific location who is not necessarily the PD nurse.

At our center, the treatment of PD-associated peritonitis follows the recommendations set out in the ISPD guidelines [9]. Initial empirical antibiotic coverage includes a combination of first-generation cephalosporin or vancomycin and third-generation cephalosporin or aminoglycoside. The antibiotic regimen is adjusted based on sensitivity and culture data. For culture-negative peritonitis that responds promptly to antibiotics, treatment with a first-generation cephalosporin or vancomycin is continued for 2 weeks. Discontinuation of a third-generation cephalosporin or aminoglycoside is at the discretion of the attending physician. However, if culture-negative peritonitis does not improve after antibiotic treatment and repeated peritoneal fluid culture remains negative, the PD catheter is removed and intravenous antibiotics are administered for 2 weeks. In cases of fungal peritonitis, we will remove the peritoneal catheter and administer systemic antifungal therapy based on the infecting organism. For patients who do not appear septic or have logistical difficulties, antibiotics are administered intraperitoneally.

## Outcomes

The primary outcome was the primary response. Secondary outcomes included complete cure, refractory peritonitis, relapse peritonitis, recurrent peritonitis, repeat peritonitis, non-repeat peritonitis, salvage antibiotics, permanent hemodialysis transfer, Tenckhoff catheter removal, and peritonitis-associated death.

### Definition of outcomes

Primary response: resolution of abdominal pain, clearing of dialysate, and peritoneal dialysate effluent neutrophil count of < 100/µL within 10 days of antibiotic treatment alone.

Complete cure: complete resolution of peritonitis without Tenckhoff catheter removal, transfer to hemodialysis, or salvage antibiotics within 120 days.

Salvage antibiotics: use of a second antibiotic regimen after the failure of the initial regimen.

Peritonitis-associated death: death of a patient with active peritonitis or death within 30 days or death during hospitalization for peritonitis.

Non-repeat peritonitis: a peritonitis episode occurring more than 4 weeks but less than 120 days after completion of therapy of a previous episode with a different organism.

Refractory peritonitis, relapse peritonitis, recurrent peritonitis, and repeat peritonitis were defined according to the ISPD guidelines [9].

### Indication for peritoneal catheter removal

The indications for peritoneal catheter removal included refractory peritonitis, relapsing peritonitis, fungal or mycobacterial peritonitis, refractory exit site and tunnel infection, and peritonitis associated with intraperitoneal pathologies, such as ruptured visceral organs, in accordance with the ISPD guidelines [9].

### Indication for permanent hemodialysis transfer

The indications for permanent hemodialysis transfer included severe sclerosis of the peritoneal membrane, inadequate dialysis despite optimal PD prescription, poor volume control despite decreased salt intake and optimized dialysis prescription, dialysate leakage, and a patient’s request for transfer to hemodialysis.

### Statistical analysis

Categorical variables are reported as frequencies and percentages, while continuous variables with normal and non-normal distributions are summarized using means with standard deviations and medians with interquartile ranges, respectively. The normal distribution was assessed using the Shapiro-Wilk test. Fisher’s exact test was used to compare categorical data between the culture-negative and culture-positive peritonitis groups, while the Mann-Whitney U test was used for continuous data with non-normal distribution. The logistic regression model was used to identify the factors associated with the primary response and culture-negative peritonitis. After univariate analysis, sex, age, culture-positive peritonitis, variables with *p*-values of < 0.1, and established risk factors for each outcome based on previous reports [14–18] were included in multivariate analysis. Statistical significance was set at a p-value of < 0.05. All statistical analyses were performed using R version 4.0.2 (R Core Team, R Foundation for Statistical Computing, Vienna, Austria, 2020).

## Results

### Population characteristics

**Fig. 1:**
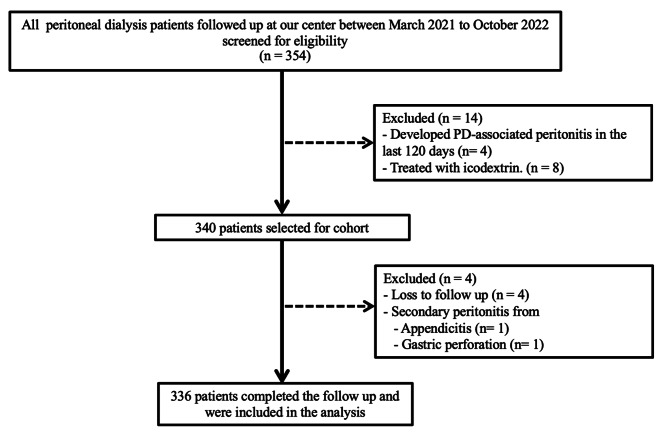
Patient selection for this prospective cohort study

From an initial pool of 354 patients who underwent screening and eligibility assessments, 340 individuals satisfied the enrollment criteria for this study. Four participants were subsequently lost to follow-up. Consequently, during the study period, which lasted from March 2021 to October 2022, a cohort of 336 patients completed the research, as represented in Fig. 1. Overall, 121 episodes of PD-associated peritonitis occurred in 81 (24%) patients, and 35 (43%) developed culture-negative peritonitis. The peritonitis rate at our center is 0.42 episodes per patient-year. Patient median age was 59 (interquartile range 43–69) years, and 45 patients (55.6%) were female. Furthermore, 77 patients (95.1%) were on continuous ambulatory PD (CAPD) and PD were performed by caregivers in 43 patients (53.1%). The baseline demographic and clinical characteristics were comparable between the two groups (Table 1), except that there were more females in the culture-negative peritonitis (CNP) group than in the culture-positive peritonitis (CPP) group and the PDF white blood cell count was lower in the CNP group than in the CPP group.

**Table 1:** Baseline demographics and clinical characteristics of patients with PD-associated peritonitis

Variable	Total (N = 81)	CNP (N = 35)	CPP (N = 46)	p-value
Age, years	59 (43–69)	55 (41–68)	63 (50–69)	0.102
Female, N (%)	45 (55.6)	25 (71.4)	20 (43.5)	0.022
DM (%)	46 (56.8)	20 (57.1)	26 (56.5)	1.000
Duration of PD, year	1.3 (0.7–3.0)	1.7 (0.8–3.5)	1.2 (0.6–2.7)	0.233
BMI, kg/m^2^	24 (21.9–27.3)	24.0 (21.7–27.3)	24.0 (22.1–27.3)	0.905
Renal diagnosis, N (%)				
Glomerulonephritis	6 (7.4)	4 (11.4)	2 (4.3)	0.396
DM	30 (37.0)	15 (42.9)	15 (32.6)	
HT	29 (35.8)	12 (34.3)	17 (37.0)	
ADPKD	5 (6.2)	2 (5.7)	3 (6.5)	
Obstructive uropathy	8 (9.9)	1 (2.9)	7 (15.2)	
Others	3 (3.7)	1 (2.9)	2 (4.3)	
PD performed by caregiver (%)	43 (53.1)	18 (51.4)	25 (55.6)	0.971
CAPD, N (%)	77 (95.1)	34 (97.1)	43 (93.5)	0.831
BUN, mg/dL	36.0 (27.0–52.0)	37.0 (26.8–50.5)	34.0 (27.0–52.0)	0.834
Creatinine, mg/dL	8.0 (5.5–11.6)	7.3 (5.2–10.7)	8.1 (5.6–13.7)	0.259
Potassium, mEq/L	3.4 (3.0–3.8)	3.3 (3.0–3.7)	3.52 (3.0–4.2)	0.227
Hemoglobin, g/dL	10.0 (8.1–11.0)	10.0 (8.7–11.5)	10.0 (7.8–10.8)	0.669
Albumin, g/dL	2.7 (1.8–3.0)	2.7 (1.8–3.0)	2.6 (1.9–3.0)	0.879
PDF WBC, ×1000/µL	2.8 (1.1–8.2)	2.5 (0.6–3.9)	3.5 (1.3–11.0)	0.024
%PMN	91 (84–95)	90 (78–95)	91 (85–95)	0.307

PD: peritoneal dialysis, CNP: culture-negative peritonitis, CPP: culture-positive peritonitis, DM: diabetes mellitus, BMI: body mass index, HT: hypertension, ADPKD: autosomal dominant polycystic kidney disease, CAPD: continuous ambulatory peritoneal dialysis, BUN: blood urea nitrogen, PDF: peritoneal dialysis fluid, WBC: white blood cell, PMN: polymorphonuclear

Continuous variables are reported as median (IQR) for non-normally distributed variables

### Culture practices

The culture practices at our center are summarized in Table 2. Compared to the CNP group, the CPP group had more PDF samples collected for culture using hemoculture bottles than using sterile tubes (82.6% vs. 51.4%, p = 0.006), had more frequent use of hemoculture than agar plates as the culture method (78.3 vs. 51.4%, p = 0.021), and had a higher proportion of patients with a PDF of > 5 ml for culture (82.6% vs. 48.6%, p = 0.003). There was a trend toward higher culture-positive results when PDF was collected at the dialysis unit. There was no significant difference in the dwell time of the PDF collected for culture, the time between the PDF being collected and sent to the laboratory, the amount of PDF discarded before collection for culture, or the number of specimens sent for culture.

**Table 2:** Comparison between culture techniques and procedures for culture-negative and culture-positive peritonitis patients

Variable	CNP N = 35	CPP N = 46	p-value
Where is the PDF collected for culture? (%)			
Ward	28 (80.0)	27 (58.7)	0.063
Dialysis unit	7 (20.0)	15 (32.6)	
Emergency department	0 (0.0)	4 (8.7)	
What is the dwell time of PDF collected for culture?			
< 1 h	0 (0.0)	1 (2.2)	0.148
1–2 h	15 (42.9)	11 (23.9)	
> 2 h	20 (57.1)	34 (73.9)	
What is the time between PDF collected and sent to laboratory?			
< 2 h	31 (88.6)	38 (82.6)	0.665
> 2 h	4 (11.4)	8 (17.4)	
Before obtaining PDF for culture, what amount of PDF was discarded from the catheter?			
< 10 mL	32 (91.4)	37 (80.4)	0.287
> 10 mL	3 (8.6)	9 (19.6)	
How was PDF collected for culture?			
Hemoculture bottle	18 (51.4)	38 (82.6)	0.006
Sterile tube	17 (48.6)	8 (17.4)	
What is the cultured method of the PDF collected?			
Hemoculture	18 (51.4)	36 (78.3)	0.021
Agar pate	17 (48.6)	10 (21.7)	
How much of PDF inoculated for culture?			
< 5 ml	18 (51.4)	8 (17.4)	0.003
> 5 ml	17 (48.6)	38 (82.6)	
Number of specimens sent for culture			
1	35 (100)	43 (93.5)	0.344
1	0(0)	3 (6.5)	

CNP: culture-negative peritonitis, CPP: culture-positive peritonitis, PDF: peritoneal dialysis fluid, hr: hour, ml: milliliter

### Organisms isolated from PDF in culture-positive peritonitis

Details of the organisms isolated from the PDF in culture-positive peritonitis are shown in Table 3. Gram-negative bacteria were the major pathogens, accounting for 76% of the episodes, with *Escherichia coli* being the most common organism (28%), followed by *Klebsiella pneumoniae* (17%) and *Pseudomonas aeruginosa* (15%). Gram-positive bacteria accounted for 18% of the episodes, of which *Streptococcus agalactiae* (4%) and *coagulase-negative Staphylococcus* (4%) were the most common organisms. The remaining episodes were caused by fungal infections (6%).

**Table 3:** Microbiological cause of culture-positive peritonitis

Organism	Total (N = 46)
Gram-negative bacteria	
*Escherichia coli*	13 (28%)
*Klebsiella pneumoniae*	8 (17%)
*Pseudomonas aeruginosa*	7 (15%)
*Enterococcus* species	2 (4%)
*Acinetobacter baumannii*	1 (2%)
*Acromobactor dentrificans*	1 (2%)
*Stenotrophomonas species*	1 (2%)
*Flavobactor* species	1 (2%)
*Sphingomonas paucimobilis*	1 (2%)
Total	35 (76%)
Gram-positive bacteria	
*Staphylococcus aureus*	1 (2%)
*Streptococcus mitis*	1 (2%)
*Streptococcus viridans*	1 (2%)
*Streptococcus agalactiae*	2 (4%)
*Coagulase-negative* *Staphylococcus*	2 (4%)
*Corynebacterium species*	1 (2%)
Total	8 (18%)
Fungus	
*Candida albicans*	1 (2%)
*Candida non-albicans*	2 (4%)
Total	3 (6%)

### Primary and secondary outcomes

The primary and secondary outcomes are summarized in Table 4. No significant difference was observed in the primary response (68.6% and 60.9% in the CNP and CPP groups, respectively; p = 0.630). Complete cure was achieved in 57.1% and 45.7% of the patients in the CNP and CPP groups, respectively (p = 0.424). The refractory peritonitis rates were 28.6% and 41.3% in the CNP and CPP groups, respectively (p = 0.342). The relapse peritonitis rates were 8.6% and 2.2% in the CNP and CPP groups, respectively (p = 0.424). The repeat peritonitis rates were 11.4% and 10.9% in the CNP and CPP groups, respectively (p = 1.000). There was no significant difference between the two groups regarding salvage antibiotics, permanent hemodialysis transfer, Tenckhoff catheter removal, or peritonitis-associated death. The recurrent and non-repeat peritonitis rates in the CPP group were 6.5% and 4.3%, respectively. In both univariate and multivariable analyses, culture-positive peritonitis, diabetes mellitus, and albumin levels were not positively associated with the primary response (Additional file 1).

**Table 4:** Primary and secondary outcomes

Endpoint	Total N = 81	CNP N = 35	CPP N = 46	p-value
		*Number (percentage)*		
Primary response	52 (64.2)	24 (68.6)	28 (60.9)	0.630
Complete cure	41 (50.6)	20 (57.1)	21 (45.7)	0.424
Refractory peritonitis	29 (36)	10 (28.6)	19 (41.3)	0.342
Relapse peritonitis	4 (4.9)	3 (8.6)	1 (2.2)	0.424
Recurrent peritonitis	3 (3.7)	N/A	3 (6.5)	-
Repeat peritonitis	9 (11.1)	4 (11.4)	5 (10.9)	1.000
Non-repeat peritonitis	2 (2.5)	N/A	2 (4.3)	-
Salvage antibiotics	37 (45.7)	14 (40.0)	23 (50.0)	0.503
Permanent hemodialysis transfer	9 (11.1)	4 (11.4)	5 (10.9)	1.000
Tenckhoff catheter removal	28 (34.6)	9 (25.7)	19 (41.3)	0.220
Peritonitis-associated death	2 (2.5)	1 (2.9)	1 (2.2)	1.000

CNP: culture-negative peritonitis, CPP: culture-positive peritonitis, N/A: not applicable

### Risk factors for culture-negative peritonitis

Univariate logistic analysis revealed that the use of an agar plate as the culture method was a predictive factor for culture-negative peritonitis (OR = 3.74; 95% CI = 1.55–9.01; p = 0.003). Additionally, hypokalemia showed a trend toward significance with an odds ratio of 3.31 (95% CI: 0.96–11.42, p = 0.058) in the univariate analysis. In the multivariate logistic analysis, two predictors for culture-negative peritonitis were identified: hypokalemia (OR = 4.88; 95% CI = 1.14–20.86; p = 0.032) and using an agar plate as the culture method (OR = 5.84; 95% CI = 1.94-17.57; p = 0.002) (Table 5).

**Table 5:** Univariate and multivariate logistic regression analyses of clinical and procedural factors for culture-negative peritonitis

Variable	Univariate analysis			Multivariate analysis		
	OR	95% CI	p-value	OR	95% CI	p-value
Age, per 1-year older	0.98	0.96–1.01	0.15	0.98	0.95–1.01	0.215
Sex category						
Male sex (ref.)	1			1		
Female sex	2.14	0.96–4.80	0.064	1.16	0.40–3.34	0.786
Albumin, per 1 g/dL	0.99	0.97–1.02	0.592			
BMI, per 1 kg/m^2^	0.99	0.91–1.07	0.777			
Potassium level						
Potassium ≥ 4 mEq/L (ref.)	1			1		
Potassium < 4 mEq/L	3.31	0.96–11.42	0.058	4.88	1.14–20.86	0.032
DM (yes/no)	1.25	0.56–2.77	0.586			
Culture method						
Hemoculture (ref.)	1			1		
Agar plate	3.74	1.55–9.01	0.003	5.84	1.94–17.57	0.002

Ref: reference, BMI: body mass index, DM: diabetes mellitus

## Discussion

This prospective cohort study demonstrated that the primary response rate in patients with culture-negative peritonitis was not different from that in patients with culture-positive peritonitis. There were also no differences between culture-negative and culture-positive patients in complete cure, refractory peritonitis, relapse peritonitis, repeat peritonitis, salvage antibiotics, permanent hemodialysis transfer, Tenckhoff catheter removal, or peritonitis-associated death. This study also showed that culture practices affect culture results. The use of a hemoculture bottle for the collection of PDF for culture, the use of hemoculture as the culture method, and the use of a PDF volume of more than > 5 ml for culture were associated with more positive culture results. The predictors of culture-negative peritonitis were identified as the use of an agar plate as the culture method and hypokalemia.

In contrast to our findings, in an observational cohort study using Australian and New Zealand Dialysis and Transplant Registry (ANZDATA) data, Fahim et al. [11] observed that compared to culture-positive peritonitis, culture-negative peritonitis was significantly more likely to be cured using antibiotics alone (77% vs. 66%) and less likely to be complicated by hospitalization (60% vs. 71%), catheter removal (12% vs. 23%), permanent hemodialysis therapy transfer (10% vs. 19%), and death (1% vs. 2.5%). Although the study used a different definition of cure to that used in our study, the authors found that culture-negative peritonitis has a more benign course. In contrast, Szeto et al. [19] identified 212 episodes of culture-negative peritonitis between 1995 and 2001 in Hongkong and found that the clinical outcomes of culture-negative peritonitis were inferior to those of representative culture-positive peritonitis treated with cefepime during the same period. The authors reported that only two-thirds of patients with culture-negative peritonitis had a primary response, and slightly more than one-third achieved a complete cure.

Our study provides valuable insights into the clinical outcomes of culture-negative peritonitis versus culture-positive peritonitis. The concept that the clinical outcomes of culture-negative peritonitis are the same at each PD center may be oversimplified. The PD center effect has contributed substantially to the appreciable variation in PD-associated peritonitis outcomes [6]. Each PD center has specific limitations, including variations in culture techniques, resources, number of PD staff, access to treatment, and antimicrobial resistance. Therefore, it may be necessary to consider the effect of the PD center on the clinical outcomes of culture-negative peritonitis, which may explain the conflicting results of previous studies. To address this, we recommend that every PD center collect data on the clinical outcomes of culture-negative peritonitis.

Another key finding is that culture practices and procedures deviating from ISPD recommendations can influence culture results. The ISPD guidelines updated in 2022 [9] recommend the use of blood culture bottles as the preferred technique for bacterial culture for PDF, the use of 5–10 ml of PDF for aerobic and anaerobic culture, a dwell time of at least 2 h before collection for culture, and a time between blood culture collection and arrival at the laboratory within 6 h. There is a still high variability in culture techniques and procedures at our center, which may result from the need for more specialized staff and the lack of culture procedure bundles. Culture techniques and procedure bundles should be implemented at each center to decrease the culture-negative peritonitis rate [20].

One potential factor contributing to the increased rate of PD-associated peritonitis in our study was hypokalemia. Existing data support a connection between hypokalemia and peritonitis [21–24], and hypokalemia was widespread in our study population, consistent with previously reported findings in Thailand [21]. The identification of hypokalemia as a predictor of culture-negative peritonitis represents a novel finding in our study. Here, we propose two possible mechanisms by which hypokalemia may contribute to culture-negative peritonitis. First, hypokalemia can induce increased intestinal permeability, facilitating the translocation of intestinal bacteria, which are predominantly anaerobic and gram-negative [25]. Given the absence of anaerobic microbe identification in our center, this translocation may have contributed to the occurrence of culture-negative peritonitis. Additionally, our study revealed a significant prevalence of Enterobacteriaceae peritonitis, aligning with prior research that identified hypokalemia as an independent risk factor for Enterobacteriaceae peritonitis [22]. Second, hypokalemia has detrimental impacts on skeletal muscle function, potentially hindering the effective execution of PD and thereby increasing the risk of contamination. An earlier study has indicated that PD patients with touch contamination are at a higher risk of developing culture-negative peritonitis than those with culture-positive peritonitis [26]. Consequently, the combination of these two factors may explain the higher prevalence of hypokalemia in culture-negative cases rather than in culture-positive ones in our study.

Because of the limited capabilities of our center to identify atypical pathogens, we suspect that some atypical pathogens may have gone undetected using our culture technique, which may have contributed to the high rate of culture-negative results. If culture-negative peritonitis is caused by unusual organisms or fastidious organisms, the outcomes could be worse compared to those of culture-positive peritonitis [27, 28]. This may explain why our study yielded different results compared to those of previous studies.

The strengths of this study include its prospective study design and detailed data collection of baseline characteristics, culture techniques, and procedures. Nonetheless, this study has several limitations. Because of the limited sample size, the differences in the clinical outcomes between culture-negative and culture-positive peritonitis could not be detected. There was a trend toward a higher complete cure rate and a lower incidence of Tenckhoff catheter removal and refractory peritonitis in the culture-negative peritonitis group than in the culture-positive peritonitis group. It is important to note that these differences may not have been statistically significant due to the small sample size of the study. The results of this study may only apply to some PD centers or countries with similar resources and settings. The elevated rate of culture-negative peritonitis observed in this study is consistent with the reported rates from other centers in Thailand, ranging between 24% and 43% [29–33]. These rates may be attributed to inadequate culture techniques and procedures. Our laboratories are equipped solely for aerobic culture, and we do not have the capability to perform sediment culture by centrifuging PDF volumes equal to or exceeding 50 mL, which has the potential to achieve a significant increase in yield [34–37].While the method used for PDF collection, the culture method for the collected PDF, and the PDF volume used for culture may confound the primary response, a multivariable analysis adjusting for culture-positive peritonitis found no effect of these variables on the association between culture-positive peritonitis and the primary response. Although the treatment protocol applied at our center was in accordance with ISPD guidelines [38], there were variations in the prescribed antibiotic combinations for empirical and subsequent treatment. However, these differences did not affect treatment response, as demonstrated in previous studies [39, 40].

## Conclusions

This study provides valuable insights into the clinical outcomes of culture-negative peritonitis compared with those of culture-positive peritonitis. However, these findings should be interpreted with caution due to the limitations of the small sample size. It is important to adhere to ISPD guideline recommendations for culture procedures. Future studies should investigate factors influencing culture-negative peritonitis outcomes, including the influence of the PD center.

### Electronic supplementary material

Below is the link to the electronic supplementary material.

Supplementary Material 1

## Data Availability

The datasets used and/or analyzed during the current study are available from the corresponding author on reasonable request.

## List of abbreviations

PD: peritoneal dialysis
PDF: peritoneal dialysis fluid
CNP: culture-negative peritonitis
CPP: culture-positive peritonitis
N/A: not applicable
OR: odd ratio
CI: confidence interval
DM: diabetes mellitus
BMI: body mass index
HT: hypertension
ADPKD: autosomal dominant polycystic kidney disease
CAPD: continuous ambulatory peritoneal dialysis
BUN: blood urea nitrogen
WBC: white blood cell
PMN: polymorphonuclear
