# Supplementary material for: Comparison of clinical outcomes between culture-negative and positive peritonitis in patients undergoing maintenance peritoneal dialysis: a prospective cohort study
Source: BMC Nephrol. 2023 Nov 17;24:340. doi: 10.1186/s12882-023-03389-7 (PMC10657137; doi:10.1186/s12882-023-03389-7)
Supplement: Supplementary file 1 — Supplementary Material 1 [file 12882_2023_3389_MOESM1_ESM.docx]

**Appendix**

**Additional file 1. Univariate and multivariate logistic regression analyses of clinical and procedural factors for primary response**

| Variable | Univariate analysis | | | Multivariate analysis | | |
| --- | --- | --- | --- | --- | --- | --- |
|  | OR | 95% CI | p-value | OR | 95% CI | p-value |
| PD fluid culture result |  |  |  |  |  |  |
| CNP (ref.) | 1 | reference |  | 1 |  |  |
| CPP | 2.03 | 0.86–4.80 | 0.121 | 2.32 | 0.85–6.33 | 0.100 |
| DM (yes/no) | 0.68 | 0.30–1.56 | 0.363 | 0.96 | 0.34–2.69 | 0.938 |
| Albumin, per 1 g/dL | 1.01 | 0.97–1.05 | 0.634 | 1.01 | 0.91–1.13 | 0.826 |
| Age, per 1-year older | 1.01 | 0.99–1.04 | 0.283 | 1.02 | 0.99–1.06 | 0.179 |
| Sex category |  |  |  |  |  |  |
| Male sex (ref.) | 1 | reference |  | 1 |  |  |
| Female sex | 0.68 | 0.30–1.56 | 0.366 | 0.68 | 0.25–1.83 | 0.446 |
| PD duration, per 1-year | 1.16 | 0.95–1.42 | 0.145 | 1.16 | 0.90–1.50 | 0.256 |

PD: peritoneal dialysis, CNP: culture-negative peritonitis, CPP: culture-positive peritonitis, DM: diabetes mellitus, ref: reference
